# Based on machine learning algorithms for estimating leaf phosphorus concentration of rice using optimized spectral indices and continuous wavelet transform

**DOI:** 10.3389/fpls.2023.1185915

**Published:** 2023-05-25

**Authors:** Yi Zhang, Teng Wang, Zheng Li, Tianli Wang, Ning Cao

**Affiliations:** College of Plant Science, Jilin University, Changchun, China

**Keywords:** continuous wavelet transform, leaf phosphorus concentration, machine learning, rice, spectral indices

## Abstract

Remotely estimating leaf phosphorus concentration (LPC) is crucial for fertilization management, crop growth monitoring, and the development of precision agricultural strategy. This study aimed to explore the best prediction model for the LPC of rice (*Oryza sativa L.*) using machine learning algorithms fed with full-band (OR), spectral indices (SIs), and wavelet features. To obtain the LPC and leaf spectra reflectance, the pot experiments with four phosphorus (P) treatments and two rice cultivars were carried out in a greenhouse in 2020-2021. The results indicated that P deficiency increased leaf reflectance in the visible region (350-750 nm) and decreased the reflectance in the near-infrared (NIR, 750-1350 nm) regions compared to the P-sufficient treatment. Difference spectral index (DSI) composed of 1080 nm and 1070 nm showed the best performance for LPC estimation in calibration (R^2 ^= 0.54) and validation (R^2^ = 0.55). To filter and denoise spectral data effectively, continuous wavelet transform (CWT) of the original spectrum was used to improve the accuracy of prediction. The model based on Mexican Hat (Mexh) wavelet function (1680 nm, Scale 6) demonstrated the best performance with the calibration R^2^ of 0.58, validation R^2^ of 0.56 and RMSE of 0.61 mg g^−1^. In machine learning, random forest (RF) had the best model accuracy in OR, SIs, CWT, and SIs + CWT compared with other four algorithms. The SIs and CWT coupling with the RF algorithm had the best results of model validation, the R^2^ was 0.73 and the RMSE was 0.50 mg g^−1^, followed by CWT (R^2^ = 0.71, RMSE = 0.51 mg g^−1^), OR (R^2^ = 0.66, RMSE = 0.60 mg g^−1^), and SIs (R^2^ = 0.57, RMSE = 0.64 mg g^−1^). Compared with the best performing SIs based on the linear regression models, the RF algorithm combining SIs and CWT improved the prediction of LPC with R^2^ increased by 32%. Our results provide a valuable reference for spectral monitoring of rice LPC under different soil P-supplying levels in a large scale.

## 1 Introduction

The fast growth of the global demand for agricultural production is increasing the chemical fertilizer application (Tilman et al., 2011; Mueller et al., 2012; Demay et al., 2023). In intensive cropping systems, phosphorus (P) fertilizer as a nonrenewable resource requires more precise management because of its different effects on yield and the environment (Sharpley and Withers, 1994; Tilman et al., 2002; MacDonald et al., 2011; Townsend and Porder, 2012). However, limiting information for regional soil P fertility status restricts the rational P management strategy development. Globally, imbalance P application within agricultural regions is increasing soil degradation with deficit application, or environmental pollution with an excessive application (Bennett et al., 2001; Carpenter, 2008; MacDonald et al., 2011; Bindraban et al., 2020). The lack of an effective method for non-destructive measurements *in situ* of P limits the holistic understanding of P requirement for crop and soil P-supplying level in a large scale. Therefore, non-destructive measurements are essential for devising precision agricultural policies and the best management practices to optimize the application of P fertilizer to improve grain yield.

As the most promising technology, hyperspectral technology can acquire variation in crop nutrient content timely and nondestructively (Takebe et al., 1990; Hansen and Schjoerring, 2003; Feng et al., 2008; Pimstein et al., 2011). Many studies have documented that leaf or canopy spectral reflectance data can be used to evaluate the nitrogen (N) status of crops, and the N deficiency influences the spectral reflectance of crops in visible region and NIR regions (Daughtry et al., 2000; Zhao et al., 2003; Xue et al., 2004; Zhao et al., 2005; Tian et al., 2014; Zhao et al., 2018). The spectral reflectance of crop leaves is known to be correlated with P status (Milton et al, 1991; Osborne et al., 2002; Yaryura et al., 2009; Pimstein et al., 2011; Mahajan et al., 2017). Generally, P deficiency promoted the visible accumulation of anthocyanin (AnC) (Jiang et al., 2007). AnC is a water-soluble pigment, which shows different colors with the change of soil P availability, and further changes the spectral reflectance of the plant (Viña and Gitelson, 2011). Compared with the spectral study of N, however, studies on crop P content are insufficient. Hence, the development of a leaf phosphorus concentration (LPC) diagnostic model by spectral reflectance technology plays an important role in precision P fertilizer management.

The spectral indices (SIs) are widely used to estimate the P concentration of crops at local, and regional scales (Mahajan et al., 2014; Mahajan et al., 2017). Many studies have shown that the SIs can be used to estimate the P concentration of wheat (Mahajan et al., 2014), litchi (Li et al., 2018a), and rice (Mahajan et al., 2017). However, the literature has shown that the relationship between the P concentration and SIs is still inconsistent. In previous studies, Mahajan et al. (2014) proposed a new normalized difference vegetation index (NDVI) of two band combinations (1080 nm, 1460 nm) for P prediction, and the correlation coefficient (R^2^) was 0.42. Mahajan et al. (2017) found that NDVI with bands at 1260 nm and 670 nm has a higher prediction accuracy of canopy P status (r = 0.67, p<0.01). Li et al. (2018a) indicated linear regression model constructed by using the ratio of reflectance difference index (RRDI_1465, 1605, 1665_) can well predict leaf P content of litchi (R^2^cv = 0.95, RMSEcv = 0.01), and the selection of sensitive bands and estimation accuracy of LPC were significantly affected by the interrelationship among LPC, pigments, and N. To ensure the performance of SIs, therefore, it is important to select the sensitive bands and suitable algorithms to create the optimized SIs models. To develop optimized SIs and improve the model accuracy of vegetation properties, considering all suitable combinations of the band based on established index formulations are widely used (Mariotto et al., 2013; Rivera et al., 2014; Yang et al., 2021b). However, due to the influence of many factors, such as different crops, growing seasons, and external environment, there is a complex nonlinear relationship between P concentration and spectral characteristics. Thus, it is still unclear whether the SIs can estimate the plant properties with high estimation accuracy (Verrelst et al., 2015; Verrelst et al., 2019). Additionally, to capture accurate and effective spectral information, continuous wavelet analysis (CWA) is becoming a promising tool for estimating biochemical constituent concentrations from leaf reflectance spectra (Cheng et al., 2011). The continuous wavelet transform (CWT) decomposes the leaf reflectance spectra into several scale components, which are composed of wavelet features as a function of wavelength and scale (Cheng et al., 2011; Li et al., 2018b). CWT has been widely used for estimating the leaf water content and nitrogen status, and was proven to be effective and have higher model accuracy compared to SIs (Cheng et al., 2011; Li et al., 2018b; Li et al., 2022).

In recent years, for modeling and analyzing crop growth and vegetation parameters, machine learning has been widely applied (Zhai et al., 2013; Heckmann et al., 2017; Wang et al., 2018; Han et al., 2019). A partial least square regression (PLSR) model was established by Chen et al. (2002) for estimating P concentration in sugarcane leaves, and the R^2^ was 0.99. Gao et al. (2019) used the support vector machine (SVM), random forest (RF), and artificial neural network (ANN) algorithms to create models for forage P content estimation, and the SVM model performed best. In addition, the coupling of SIs with machine learning algorithms can improve the accuracy obviously in crop parameter estimation, such as leaf water content (Zhang et al., 2021), and above-ground biomass (Wang et al., 2016; Yang et al., 2021b). The input variables of machine learning can be optimized by using the SIs, such as dimension and multicollinearity reduction (Yang et al., 2021b). However, the previous studies showed the different performances of various models. Therefore, selecting suitable input variables to feed machine learning algorithms is critical for estimating rice LPC.

Previous studies have investigated the full spectrum and feature bands as input variables for machine learning algorithms to estimate the crop LPC. However, limited studies reported the sensitive bands, optimized SIs, and spectral transformation techniques coupling with machine learning algorithms in the estimation of rice LPC. To improve modeling precision and dimension reduction for rice LPC, therefore, there is a need to combine spectral index, wavelet analysis, and machine learning algorithms. In this study, we applied the rice leaf reflectance under different P application rates and explored the optimal prediction model for LPC by using five machine learning algorithms fed with full-band, spectral indices, and continuous wavelet features. This research aimed to provide a basic reference for LPC spectral monitoring of rice under different soil P-supplying levels in a large scale. The specific objectives were (1) to evaluate the performance of SIs and CWT of original spectrum in estimating rice LPC and (2) to compare the full-band, optimized results of SIs and CWT coupled with five machine learning algorithms in predicting rice LPC.

## 2 Materials and methods

### 2.1 Experimental design and growth conditions

The pot experiments of rice were carried out in the greenhouse of Inner Mongolia Agricultural University (111°42′ E, 40°48′ N) during 2020-2021 in Hohhot, Inner Mongolia, China. The air temperature and humidity in the greenhouse were maintained at 25-28 °C and 60-70%. The photoperiod was 12h light and 12h dark per day (LD 12:12) in white fluorescent light (about 150 µmol/m²/s).

Pot experiments with four P treatments, which are 0, 20, 40, and 80 kg P_2_O_5_ ha^-1^, respectively (P0, P1, P2, and P3), and two rice cultivars (Longjing 31 and Wuyoudao 4) were conducted. The pot size was 40 × 20 × 20 cm. The experiment was a randomized complete block design with ten replicates. Soil pH, organic matter, total N, total P, available N, and available P were 7.8, 17.1 g kg^-1^, 0.67 g kg^-1^, 0.31 g kg^-1^, 29.8 mg kg^-1^, and 8.9 mg kg^-1^, respectively. Phosphorus fertilizer applications such as monopotassium phosphate were performed before sowing.

### 2.2 Spectral data collection

**Figure 1:**
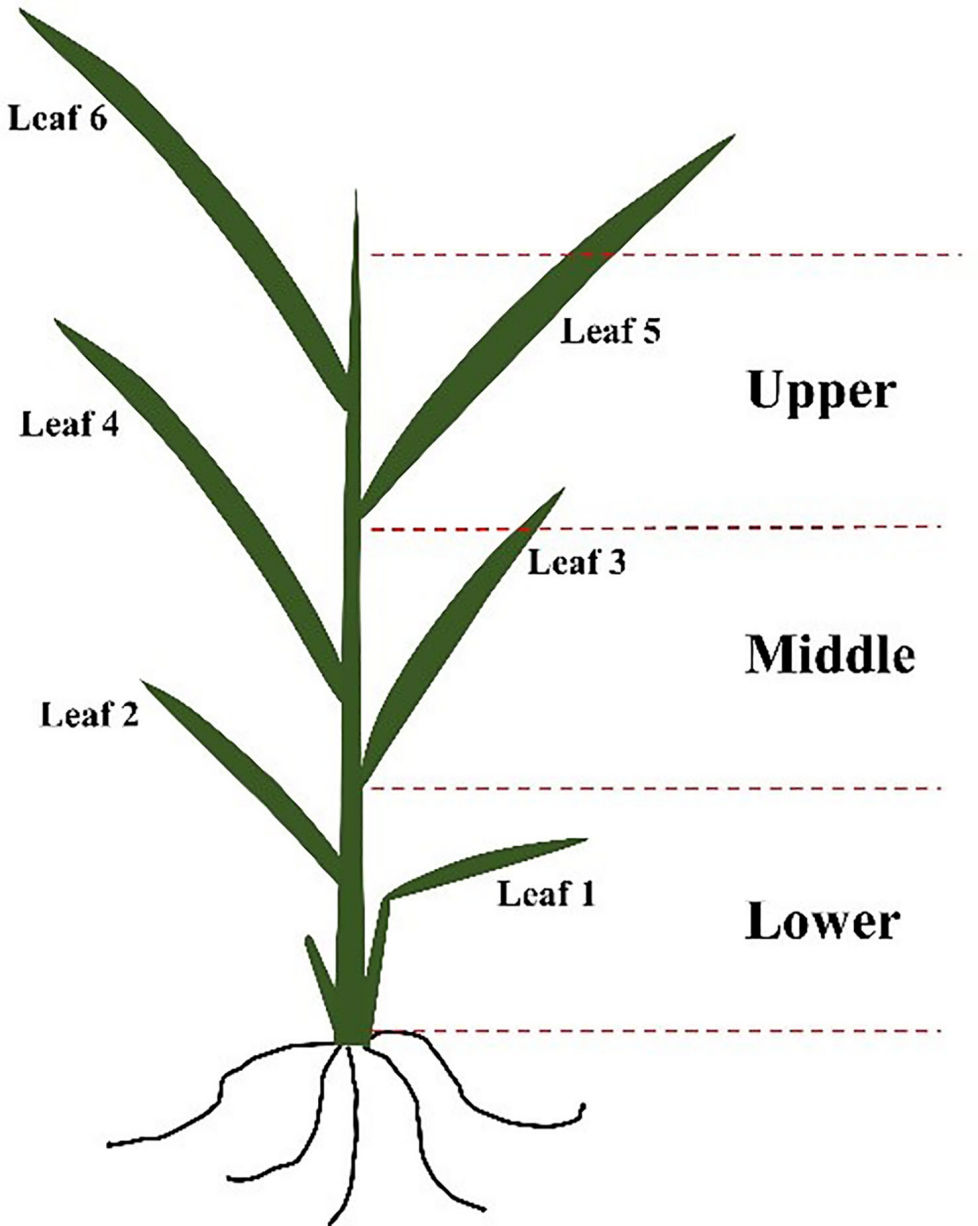
Diagram of different layers of rice leaf.

The spectral reflectance of rice leaves in the upper, middle, and lower layers (**Figure 1**) were measured at the critical stage of P nutrition (tillering stage with six leaves) using a ground object spectrometer PSR+3500 (Spectral Evolution Inc., Lawrence, MA, USA). This instrument records reflectance between 350-2500 nm with a sampling interval of 1 nm and spectral resolution of 3 nm@700 nm, 8 nm@1500 nm, and 6 nm@2100 nm respectively. Output data were composed of the reflectance of 2151 spectral channels. Before measuring, flip the leaf clip and calibrating with the whiteboard in the pistol grip. Put the leaf into the leaf clip during measurement. The observation angle was 90°, the area of view was about 0.5 cm^2^ and all spectral measurements were measured between 11:30 a.m. to 2:00 p.m. on clear sunny days (Darvishzadeh et al., 2008; An et al., 2020). Each leaf was measured with three replicates, and the average value was taken as the spectral reflectance of the rice leaf.

### 2.3 Plant sampling and LPC measurements

After spectral data collection, rice leaves in the same layer were collected for measuring leaf dry mass and LPC. All plant samples were oven-dried at 105 °C for 0.5 h and then dried at 75 °C until a constant weight was reached for biomass measurements. After calculating the biomass, the samples were ground to a fine powder (0.25 mm sieve) and the molybdate-blue colorimetric method was used for determining the LPC (mg g^−1^) of each sample (Murphy and Riley, 1962).

A total number of 456 rice leaf samples were collected during the 2 years of the experiment. The pooled data were divided randomly into an independent calibration dataset (70% of the pooled data, 319 samples) and a validation dataset (30% of the pooled data, 137 samples). The calibration dataset was used to establish the models, and the validation dataset was used to validate the models.

### 2.4 Spectral indices and continuous wavelet transform analysis

#### 2.4.1 Spectral indices (SIs)

A large number of SIs have been created to estimate the nutrition parameters of crops. Especially the two-band SIs including ratio spectral index (RSI), difference spectral index (DSI), and normalized differential spectral index (NDSI) are the most classic SIs algorithms (Jordan, 1969; Rouse et al, 1974; Tucker, 1979). The calculation formula of these SIs are shown as follows.


(1) $$\begin{array}{c} RSI=\frac{R_{\lambda 1}}{R_{\lambda 2}} \end{array}$$

(2) $$\begin{array}{c} DSI=R_{\lambda 1}-R_{\lambda 2} \end{array}$$

(3) $$\begin{array}{c} NDSI=\frac{R_{\lambda 1}-R_{\lambda 2}}{R_{\lambda 1}+R_{\lambda 2}} \end{array}$$

*R_λ1_ and R_λ2_* represent the reflectance of any two single bands in the range of 350-2500 nm, respectively, and a self-developed code in MATLAB R2021b software (The MathWorks Inc., Massachusetts, USA) was used to select the bands. The relationships between rice LPC and three SIs were analyzed for determining the optimal estimation model of LPC.

#### 2.4.2 Continuous wavelet transform (CWT) analysis

CWT is a signal analysis and processing tool which can realize multi-frequency and multi-scale decomposition of spectral information. It decomposes the signal into a series of wavelet functions obtained by the same wavelet basis function. The component in each scale can be directly compared with the input data of spectral reflectivity. At the same time, more valuable spectral information can be obtained (Rivard et al., 2008; Cheng et al., 2011). Usually, choosing the appropriate wavelet function is the primary task of the transform process. In this study, fifteen wavelet functions in MATLAB R2021b were used and ten scales were calculated for each wavelet function. The Mexican Hat (Mexh) wavelet functions smooth the spectral data with the Gaussian function and then calculate the second derivative. It can filter and denoise spectral data effectively (Singh et al., 2013). According to the results of R^2^ between wavelet functions and the LPC of rice, the transformation effect based on the Mexh function produced the highest model accuracy. Therefore, Mexh was selected as the basic function of CWT in this study and was realized in MATLAB R2021b.

### 2.5 Machine learning algorithms

#### 2.5.1 Partial least squares regression (PLSR)

PLSR is that the eigenvalues are reduced to a small group of unrelated features through a certain operation process, and the least square regression method is performed on these features, which can solve the problems of multi-collinearity between features and feature dimension greater than the sample numbers (Ramadan et al., 2005). In this study, the PLSR program was applied using Python (version 3.7.0, The Python Software Foundation, USA) software, and the parameters were the default settings.

#### 2.5.2 Least absolute shrinkage and selection operator (LASSO)

LASSO is a biased estimation algorithm for solving multiple collinear problems (Tibshirani, 2011). Its basic principle is to add L1 regularization constraints to the parameters based on conventional linear regression, to simplify the refined model and prevent over-fitting of the model. The LASSO program was conducted using Python software, and the selection parameter was set to ‘cyclic’, which means that the update of the regression coefficient in each iteration is based on the last operation.

#### 2.5.3 Random forest (RF)

The RF regression model is based on the decision tree, random attributes are introduced to construct an integrated evaluator (Breiman, 2001). Each decision tree learns independently and predicts independently. The prediction results are determined by averaging over all the trees (Liaw and Wiener, 2002; Hao et al., 2015; Yang et al., 2021b). In this paper, the RF program was applied using Python software, and the parameters were the default settings.

#### 2.5.4 Support vector machine (SVM)

SVM is based on the structural risk minimization principle and statistical learning theory, which is suitable for machine learning of small samples (Cortes and Vapnik, 1995). In this study, the kernel function selected when using SVM is the radial basis kernel function (Radial Basis Function), which is suitable for solving partial nonlinear problems. The SVM program was applied using Python software, and the parameters were the default settings in this study.

#### 2.5.5 Back propagation artificial neural network (BPANN)

As an artificial intelligence method, BPANN uses an error backpropagation algorithm to obtain the multilayer feedforward neural network (Ramadan et al., 2005). It has a strong nonlinear fitting ability and is widely used. BPANN program was conducted using Python software, and the parameters were the default settings.

**Figure 2:**
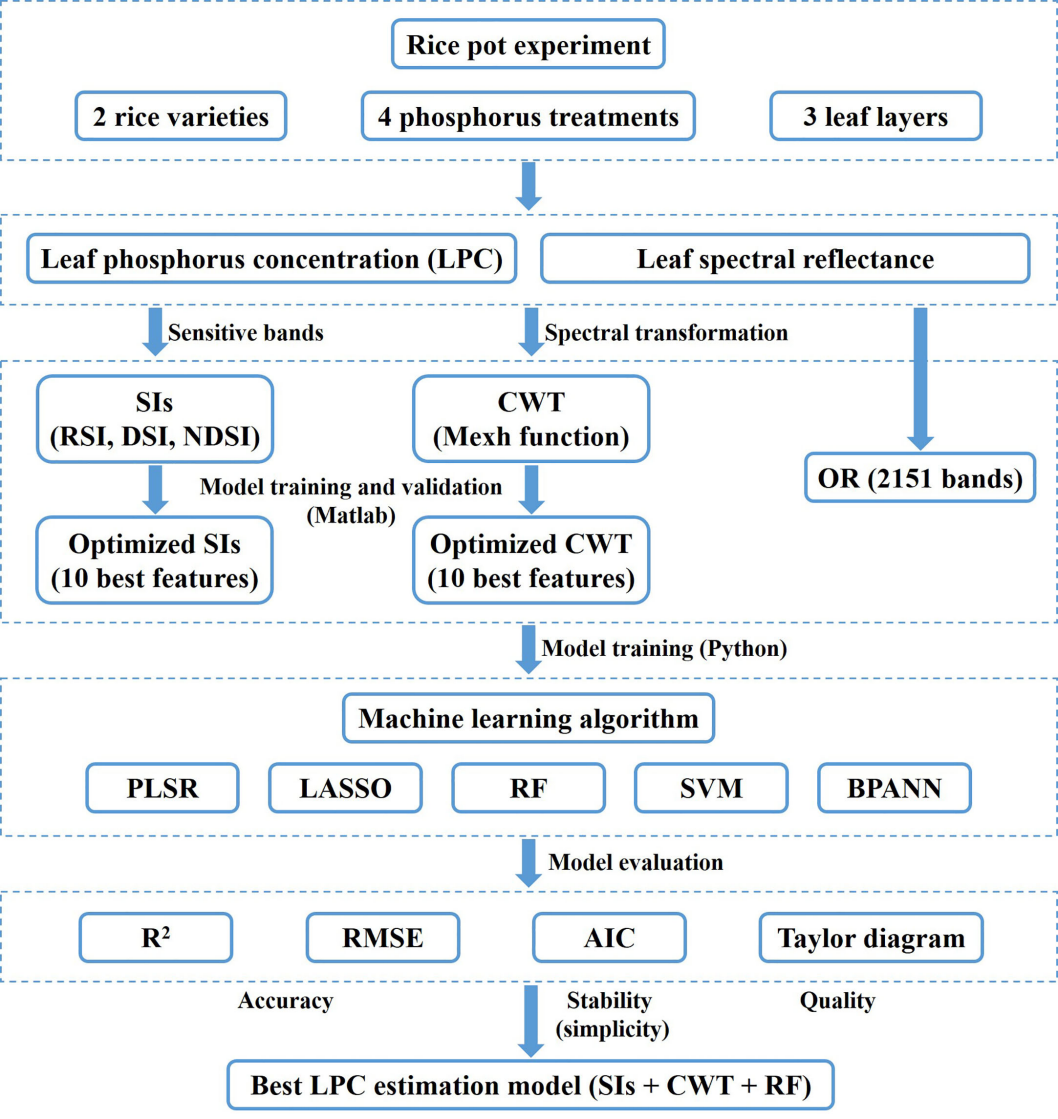
Flowchart of the methodology.

The LPC of rice was taken as the dependent variable. The independent variables were the original full band (all 2151 bands ranging from 350-2500 nm, OR), optimized SIs (10 best features), optimized CWT (10 best features), and the combination of SIs and CWT (20 input features, SIs + CWT), respectively. And then the PLSR, LASSO, RF, SVM, and BPANN models were established. A flowchart of the rice LPC estimation model construction is shown in **Figure 2**.

### 2.6 Model accuracy evaluation

The accuracy and simplicity of the model were evaluated by the determination coefficient (R^2^), root mean square error (RMSE, mg g^−1^), and Akaike information criterion (AIC). The calculation formula is shown as follows:


(4) $$\begin{array}{c} R^{2}=1-\frac{\sum_{i=1}^{n}{\left(y_{i}-x_{i}\right)}^{2}}{\sum_{i=1}^{n}{\left(x_{i}-\bar{x}\right)}^{2}} \end{array}$$

(5) $$\begin{array}{c} RMSE=\sqrt{\frac{\sum_{i=1}^{n}{\left(y_{i}-x_{i}\right)}^{2}\;}{n}} \end{array}$$

(6) $$\begin{array}{c} AIC=2k+n*\ln\left(\frac{\sum_{i=1}^{n}{\left(y_{i}-\bar{x}\right)}^{2}}{n}\right) \end{array}$$

where $\bar{x}$ represents the average of measured values. *x_i_* and *y_i_* represent the measured values and predicted values of LPC, respectively. *n* is the number of samples, and *k* is the number of features. The smaller RMSE with larger R^2^ values means better model estimation accuracy. AIC is an index for evaluating the model complexity, and the smaller value means a lower risk of overfitting.

Cross-validation can evaluate the machine learning model skills, which have a lower bias than other methods. The 10-fold coefficient of variation generally attains the lowest mean squared error and variance (Gao et al., 2019). For evaluate the model performance, the coefficient of determination (R^2^) and root mean squared error (RMSE) of the ten iterations were calculated in this study. Higher R^2^ and smaller RMSE indicate that the model has higher accuracy.

Taylor diagram provides a visual framework for the comparative assessment of different model results. The diagram can also be used to quantify the degree of correspondence between the predicted value of the models and the observations. It uses three statistics, the Pearson correlation coefficient, RMSE, and standard deviation (amplitude of variations) between predicted and observed values (Taylor, 2001). In this study, the Taylor diagram was used to evaluate the accuracy of the LPC estimation models based on the machine learning algorithms.

### 2.7 Statistical analysis

A one-way ANOVA was used to compare the means of LPC among different rice varieties, leaf layers, and P treatment based on the least significant difference at a 0.05 level of probability with DSS Statistics.

## 3 Results

### 3.1 Variations in LPC and spectral reflectance

**Figure 3:**
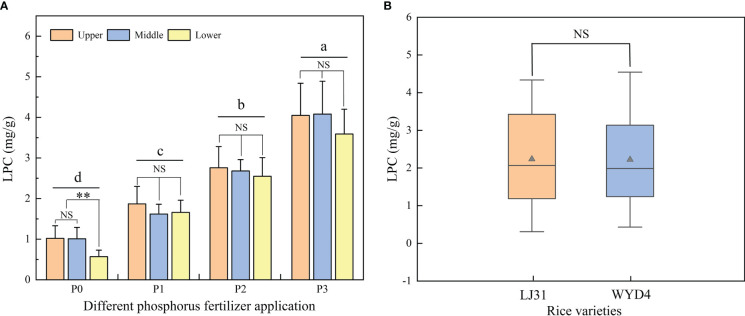
Comparison of LPC in different **(A)** P treatment and leaf layers, **(B)** rice varieties. Different letters above the bars are significantly different in different P treatments (P< 0.05). NS and ** indicate no significant difference and significance at P< 0.01.

**Figure 3A** shows the rice LPC in different P fertilizer applications, there was a significant difference among different P treatments. And the variation trend of LPC was P3 > P2 > P1 > P0. In terms of different leaf layers (**Figure 3A**), the rice LPC decreased from the upper to the lower layer, and there was no significant difference except for the P0 treatment. The effect of the P application rate on the spectral reflectance of rice leaves in Longjing 31 (LJ31) and Wuyoudao 4 (WYD4) were analyzed, and there was no significant difference between the two rice varieties (**Figure 3B**).

**Figure 4:**
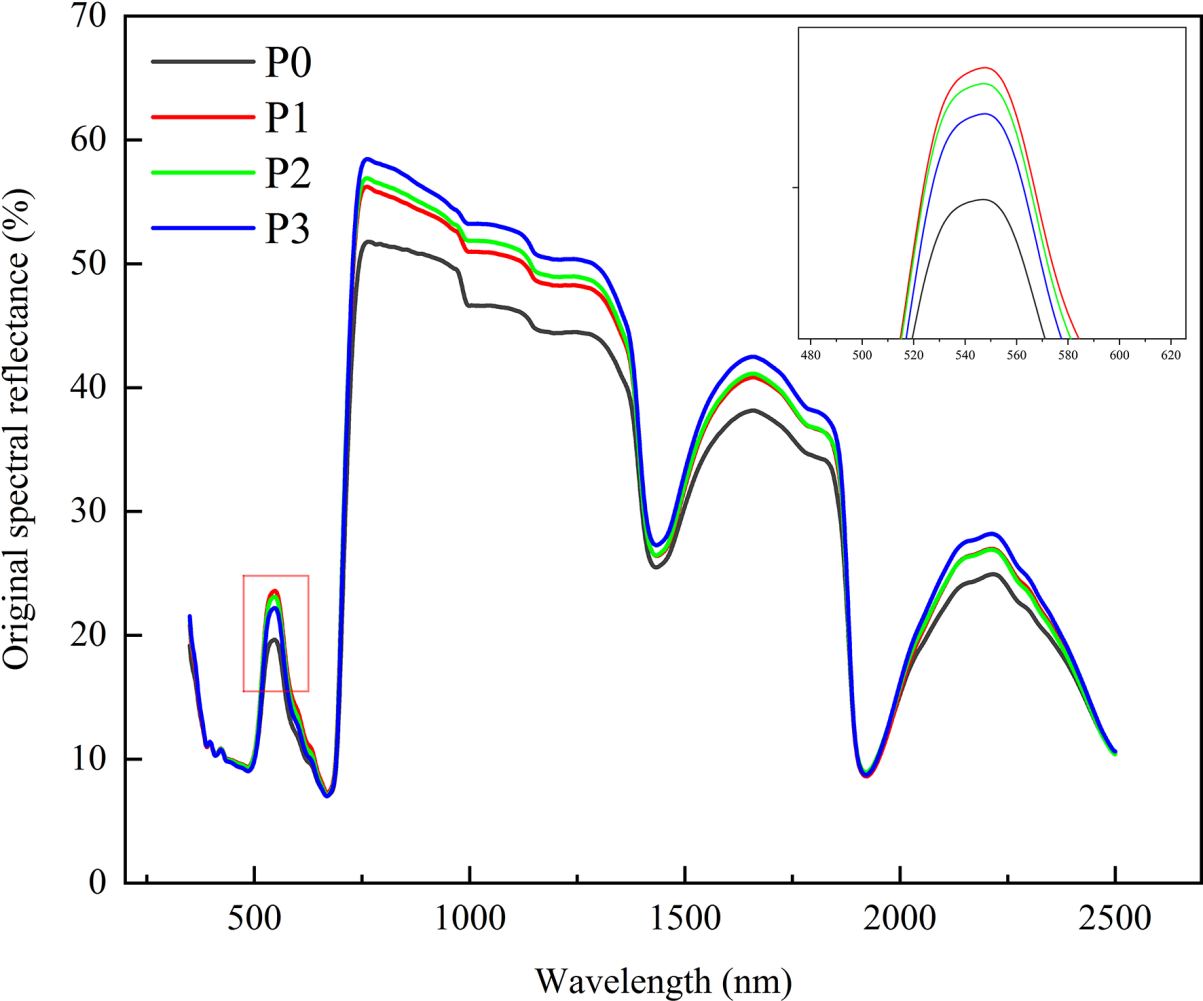
Original spectral reflectance of rice leaves in different P treatments.

**Figure 5:**
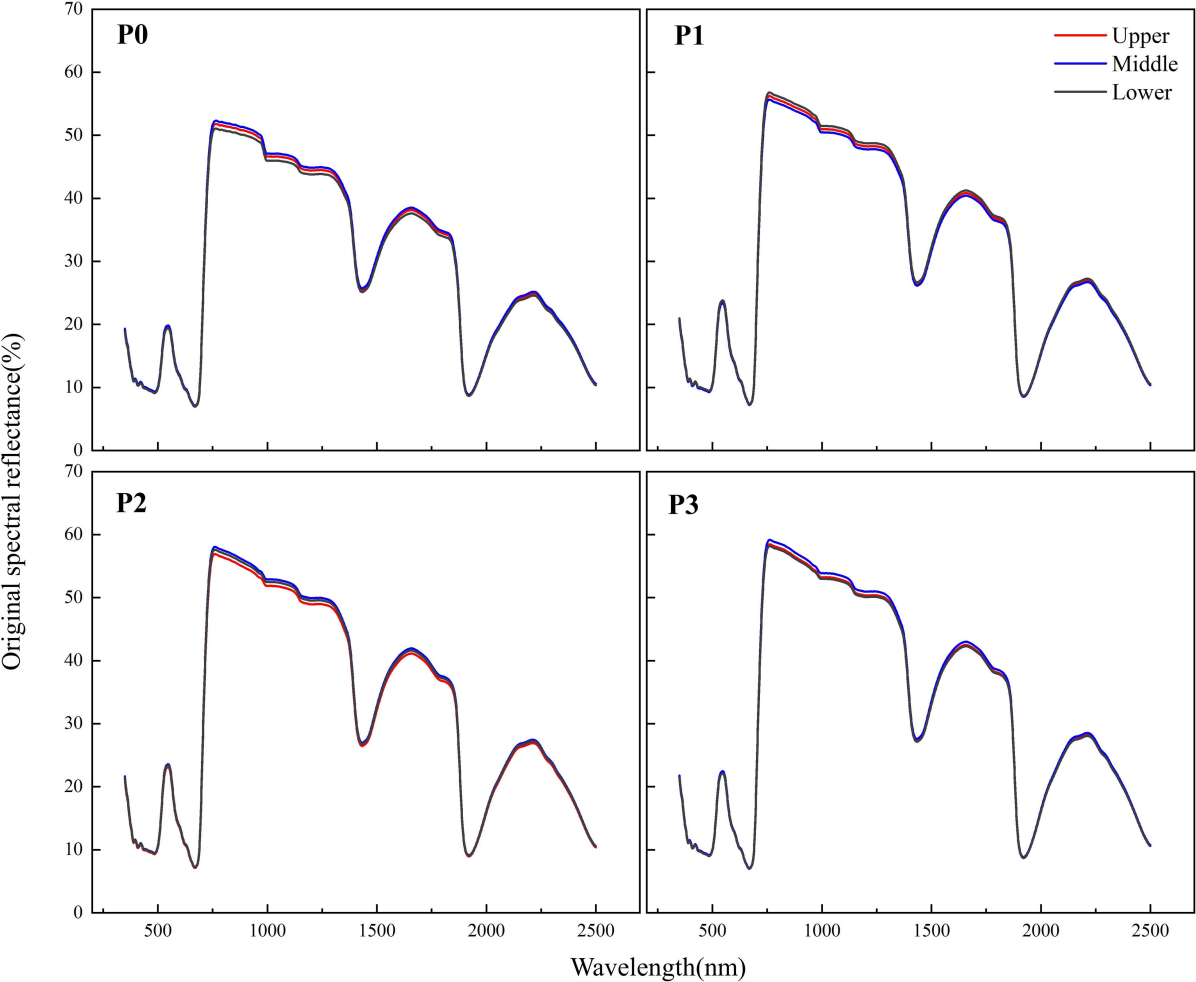
Original spectral reflectance of rice leaves in different leaf layers.

**Figure 4** shows the original spectral reflectance of rice leaves in different P treatments in the range of 350-2500 nm. The results showed the P application rate significantly affected the leaf reflectance spectra, and the effects were different in the visible region (350-750 nm) and NIR regions (750-1350 nm). The spectral reflectance of rice leaf was at a low level (25%) in the visible region. The P deficiency mainly increased the leaf reflectance (P1 > P2 > P3) at 550 nm. In the NIR regions, in contrast to the visible region, the leaf spectral reflectance was higher, and the P deficiency decreased leaf reflectance (P3 > P2 > P1 > P0). **Figure 5** shows the original spectral reflectance of rice leaves in different layers. The results showed there was no difference in spectral reflectance between the three layers. Thus, all rice leaf data in different layers were pooled into one data set, and randomly allocated for model training and testing.

### 3.2 Estimation of rice LPC using spectral indices

**Figure 6:**
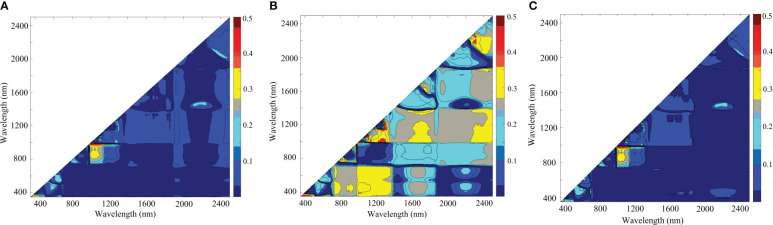
Contour maps of the determination coefficient (R^2^) between LPC and **(A)** RSI, **(B)** DSI, and **(C)** NDSI values.

To understand the relationships between LPC and RSI, DSI, and NDSI, the contour maps of the determination coefficient (R^2^) between three SIs and LPC were plotted in **Figure 6**. As illustrated, the performance of RSI was almost the same as NDSI, and the sensitive regions were mainly located in the NIR regions. The “hot spot” occurred in the area of the combination of 980-1140 nm (horizontal axis) and 960-990 nm (vertical axis). The R^2^ for the relationships between LPC and RSI, NDSI in the ranges were higher than 0.4. The sensitive band ranges for DSI were mainly concentrated on 1100-1400 nm (horizontal axis) and 1000-1300 nm (vertical axis). Overall, DSI consisting of 1089 nm and 1070 nm is the best performing spectral index for the estimation of LPC.

**Figure 7:**
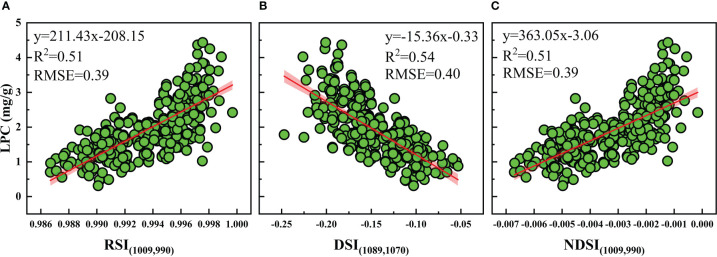
The relationships between LPC and optimized **(A)** RSI, **(B)** DSI, and **(C)** NDSI for the calibration dataset.

**Figure 8:**
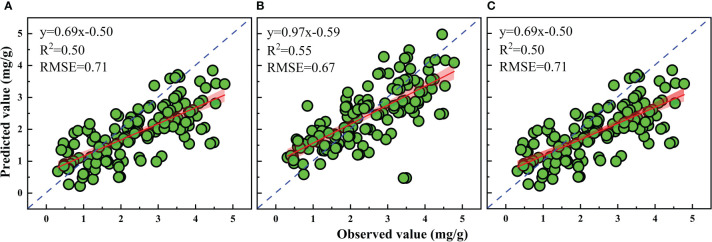
Validation of the estimation models for LPC based on optimized **(A)** RSI, **(B)** DSI, and **(C)** NDSI.

Based on the best performing SIs, rice LPC was estimated. The best correlations with LPC were selected to construct the traditional linear regression models (**Figure 7**). The results showed that the DSI (1089, 1070 nm) had higher R^2^ (0.54) in different calibration datasets compared to the RSI (1009, 990 nm) and NDSI (1009, 990 nm). The models were validated by the validation dataset. Relationships between the observed data and the predicted value of LPC by using the three SIs were illustrated in **Figure 8**. The results showed that the DSI had the best performance with an R^2^ of 0.55 and RMSE of 0.67 mg g^−1^ compared to RSI and NDSI. Therefore, the changes in LPC caused by different P supply levels can be estimated by optimized spectral index (DSI). However, the estimation accuracy of the linear regression models based on SIs was not high, and the calibration R^2^ lower than the validation R^2^. These results showed the SIs models were underfitting and unstable.

### 3.3 Estimation of rice LPC using continuous wavelet transform

**Figure 9:**
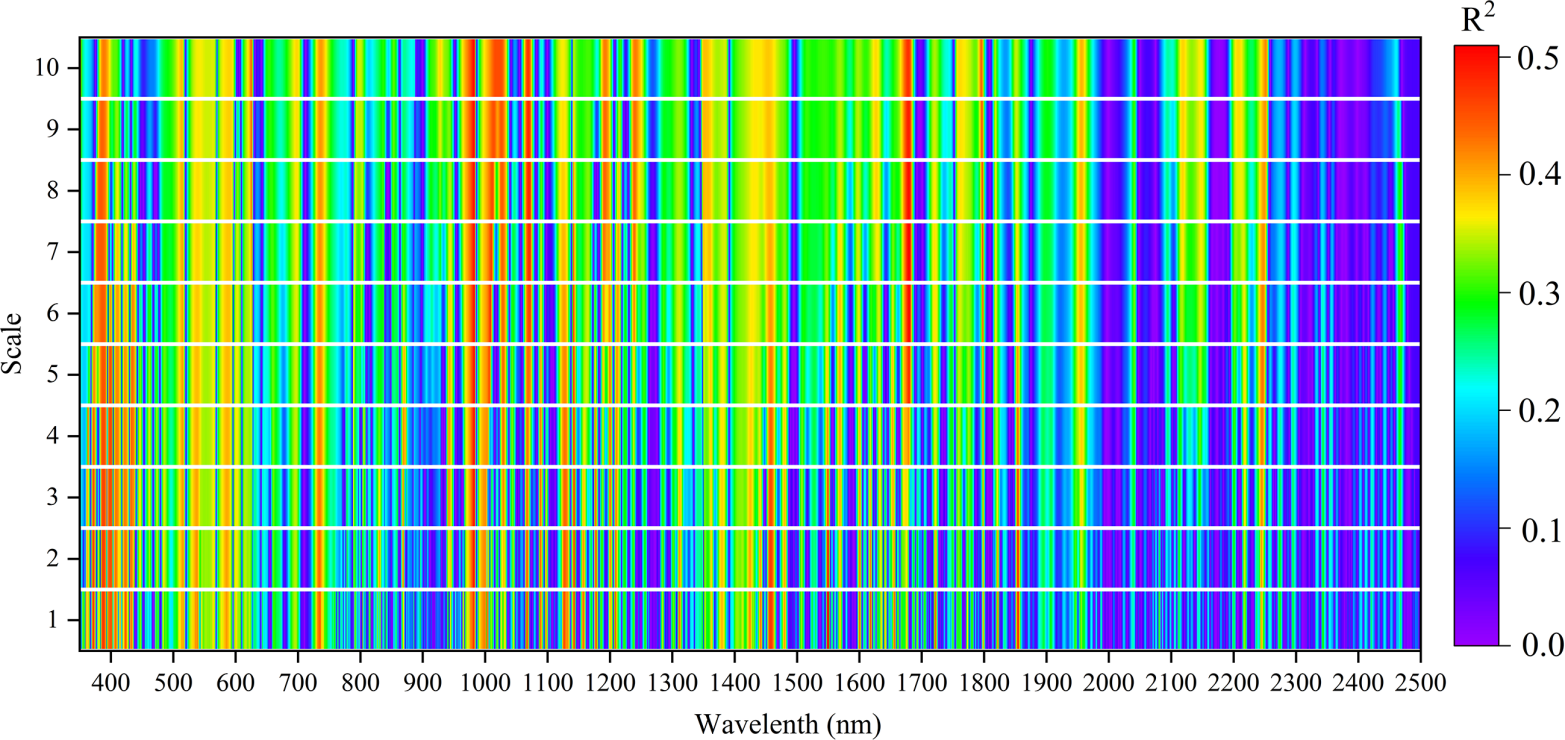
Correlations between CWT and LPC at different transform scales.

**Figure 9** shows the relationships between using CWT of reflectance spectra on ten scales based on Mexh function and LPC of rice. Between 400 and 1700 nm, four wavelet features were observed that strongly correlated with the LPC of rice. The feature regions were centered at 400 nm, 1000 nm, 1470 nm, and 1680 nm. An optimal wavelet feature was selected on each scale to construct the LPC estimation model. The wavelet feature at 1680 nm and scale 6 provided the strongest correlation, with calibration R^2^ of 0.58, validation R^2^ of 0.56, and RMSE of 0.61 mg g^−1^ (**Table 1**). These results represent that the R^2^ values are improved by using CWT analysis compare with SIs (validation R^2^ = 0.55).

**Table 1:** Calibration and validation of LPC estimation models based on continuous wavelet function (Mexh).

Feature (wavelength in nm, scale)	Calibration dataset		Validation dataset	
	Fitted equation	R^2^	R^2^	RMSE
Mexh (1550 nm, 1)	y = 647.56x + 1.80	0.50	0.48	0.73
Mexh (982 nm, 2)	y = -36.31x + 2.99	0.51	0.49	0.71
Mexh (983 nm, 3)	y = -16.51x + 2.55	0.50	0.47	0.73
Mexh (982 nm, 4)	y = -6.70x + 3.14	0.50	0.50	0.70
Mexh (982 nm, 5)	y = -4.25x + 3.15	0.50	0.49	0.71
Mexh (1680 nm, 6)	y = 22.74x + 0.15	0.58	0.56	0.61
Mexh (1679 nm, 7)	y = 15.42x - 0.06	0.53	0.52	0.66
Mexh (1679 nm, 8)	y = 11.69x - 0.20	0.52	0.51	0.69
Mexh (982 nm, 9)	y = -1.88x + 2.92	0.51	0.50	0.71
Mexh (982 nm, 10)	y = -1.72x + 2.80	0.52	0.52	0.67

### 3.4 Estimation of rice LPC using machine learning algorithms

**Figure 10:**
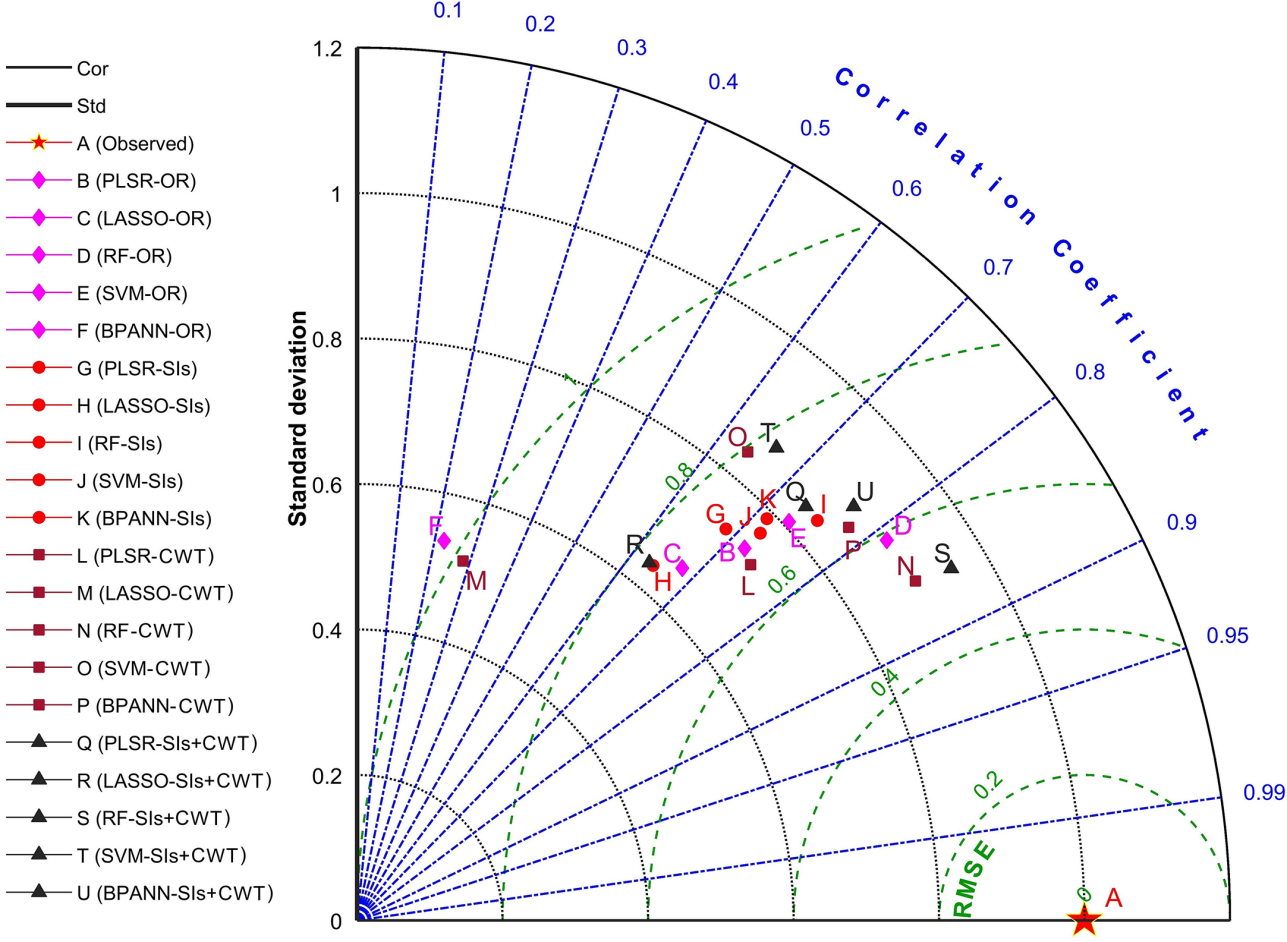
Precision comparison of the 20 LPC estimation models based on Taylor diagram.

**Figure 10** shows the statistical comparison results between 20 estimation models and the observations. The models constructed using RF - CWT (point N) and RF – SIs + CWT (point S) were closer to the observation data (point A) on the Taylor diagram, and thus these two models are relatively superior to the other models. And the standard deviation of RF – SIs + CWT was closer to 1, which means the model has the best prediction performance. The accuracy of the 20 models for rice LPC was evaluated with 10-fold cross-validation (**Table 2**). The result indicates that the RF algorithm fed with the combination of SIs and CWT (RF – SIs + CWT) significantly improved estimation accuracy. In the validation set, R^2^ and RMSE were 0.73 and 0.50 mg g^−1^, respectively and the model presents the lowest AIC of -3402.43 (**Table 2**).

**Table 2:** 10-fold cross-validation results of machine learning models.

Variables	No. of bands or features	Models	Calibration dataset	Validation dataset		AIC
			R^2^	R^2^	RMSE	
OR	2151	PLSR	0.49	0.52	0.72	1210.55
		LASSO	0.47	0.46	0.76	1259.86
		RF	0.95	0.66	0.60	1044.27
		SVM	0.52	0.54	0.70	1184.86
		BPANN	0.48	0.50	0.96	1472.91
SIs	10	PLSR	0.45	0.47	0.73	-3058.87
		LASSO	0.43	0.41	0.77	-3010.22
		RF	0.81	0.57	0.64	-3178.87
		SVM	0.53	0.52	0.68	-3123.58
		BPANN	0.48	0.51	0.68	-3143.67
CWT	10	PLSR	0.59	0.55	0.67	-3137.09
		LASSO	0.12	0.08	0.95	-2718.64
		RF	0.97	0.71	0.51	-3385.95
		SVM	0.50	0.43	0.77	-3010.22
		BPANN	0.74	0.61	0.62	-3207.83
SIs + CWT	20	PLSR	0.57	0.54	0.67	-3117.09
		LASSO	0.42	0.40	0.78	-2978.49
		RF	0.95	0.73	0.50	-3402.43
		SVM	0.51	0.44	0.77	-2990.22
		BPANN	0.73	0.59	0.62	-3187.83

## 4 Discussion

Rice growth is directly affected by soil P-supplying levels (Schachtman et al., 1998; Shen et al., 2011; Jiang et al., 2021). As an important indicator of crop growth, the changes in LPC can be obtained by spectral sensing technology. Previous research has discovered that various crops have varied P spectral response characteristics (Milton et al, 1991; Yaryura et al., 2009; Pacumbaba and Beyl, 2011). Our study measured the rice leaves in three layers at the tillering stage. The results showed the rice LPC decreased from the upper to the lower layer, and there was a significant difference between the upper and the lower layer in the P0 treatment. These results demonstrated the P would transfer from old leaves to new leaves when rice is suffered from extreme P deficiency. Previous studies indicated that P remobilization from aging organs to young organs occurred generally during the late vegetative and reproductive growth of plants (Veneklaas et al., 2012; Wang et al., 2021). In this study, the leaf samples were taken at the middle vegetative growth of rice, so there was no significant difference among the three layers under other P treatments. And the P deficiency decreased all rice leaves reflectance in the NIR regions (750-1350 nm), which is similar to the findings of Pacumbaba and Beyl (2011). In addition, many studies have investigated the N nutrition of plants, the sensitive bands of crop N concentration range from 340 nm to 900 nm (Li et al., 2014; Yang et al., 2021a). P concentration of the crop was slightly different from the N, the sensitive bands of crop P concentration were located from the visible region to NIR regions (Osborne et al., 2002; Yaryura et al., 2009; Ramoelo et al., 2011; Mahajan et al., 2014). In our study, the sensitive bands of LPC were located in the NIR regions (750-1350 nm).

**Figure 11:**
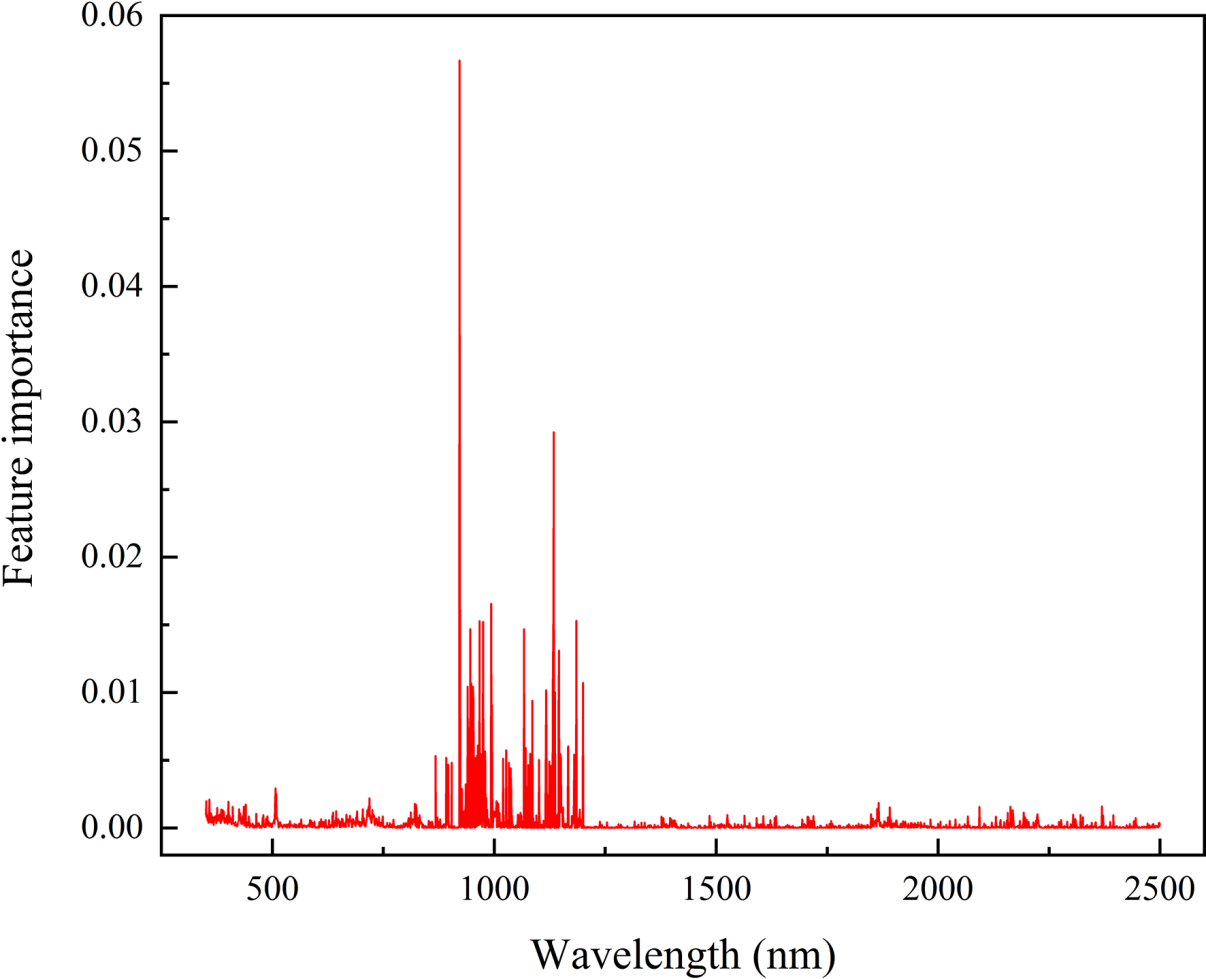
RF model feature importance score based on full spectrum.

In general, N deficiency increases the leaf reflectance in green and red edge areas, which is due to the decrease of chlorophyll content in leaves (Daughtry et al., 2000; Zhao et al., 2003; Zhao et al., 2005). In P deficiency, one of the characteristic responses of plants is the visible accumulation of anthocyanin (AnC) (Jiang et al., 2007). Existing studies suggested that the AnC spectral feature of plant leaves was peaking around 550 nm in the visible region, and the spectral reflectance of AnC increased sharply near 700nm (Gitelson et al., 2001; Liu et al., 2015; Wang et al., 2020). Moreover, the peak magnitude was closely related to the content of AnC (Gitelson et al., 2001), and also with the increasing of AnC content, the reflectivity of leaves decreased (Liu et al., 2015). The AnC spectral features of plant leaves are similar to our results, which the leaf reflectance decreased with increasing P application rate in the visible region. Therefore, we considered that the spectral reflectance of P is affected by the AnC content of leaves in the visible region. Several studies found that the green (540-560 nm) and red (640-760 nm) bands were sensitive regions to AnC in plant leaves (Gitelson et al., 2006; Merzlyak et al., 2008; Liu et al., 2015; Wang et al., 2020). In contrast, our results showed the NIR regions (990 nm, 1009 nm, 1070 nm, and 1089 nm) were important to LPC estimation in rice by using SIs. In the optimal CWT, the sensitive bands also were 982 nm, 983 nm, 1550 nm, 1679 nm, and 1680 nm. And according to the feature importance of the RF model (**Figure 11**), 922 nm, 1134 nm, 983 nm, 923 nm, and 1185 nm were the sensitive bands for rice LPC estimation. The results are similar to the findings of other crops, the NIR was the best sensitive region for P estimation. For example, Ramoelo et al. (2011) indicated that the spectral absorption features used for P estimation of forage were mainly located in the NIR regions. Mahajan et al. (2014) found that the combination of reflectance in NIR and shortwave infrared (SWIR) regions significantly improved the accuracy of P content prediction of wheat. Therefore, NIR regions are more suitable for predicting the LPC of rice at tillering stage.

CWT has significant advantages in effectively obtaining spectral information, denoising, and dimensionality reduction of hyperspectral data (Ebrahimi and Rajaee, 2017; Li et al., 2022). Some previous studies confirmed CWT increased the estimation accuracy of crop leaf nitrogen status in rice, wheat, and summer maize (Li et al., 2018b; Li et al., 2022). Moreover, the Mexh wavelet family is often used as a CWT analysis method. Singh et al. (2013) found that in the quantification of crop leaf pigments, the model obtained by using the Mexh wavelet family has the highest accuracy compared with original spectra and other transformations of spectral reflectance data (Singh et al., 2013). Our study also found that the coefficient of correlation between the spectral data and rice LPC was improved by the CWT (Mexh function) of the original spectral data.

Machine learning methods have also been applied to predict the crop growth information and vegetation parameters, such as leaf water content (Zhang et al., 2021), and above-ground biomass (Wang et al., 2016; Yang et al., 2021b) to further improve the accuracy of modeling. The estimation accuracy is affected by crop species, vegetation parameters, spectral index, and the type of machine learning algorithm (Chen et al., 2002; Gao et al., 2019). Previous studies showed the different performances of various algorithms. In the current study, PLSR, LASSO, RF, SVM, and BPANN algorithms were used to estimate the rice LPC. The effects of the five machine learning algorithms were different, and the four input variables (OR, SIs, CWT, and SIs + CWT) had a great influence on the estimation effect of the models. The numbers of input features of the models coupled with SIs and CWT were significantly less than that of OR, but the accuracy was improved. The results mean that the dimensionality reduction of input variables is crucial for machine learning algorithms (Yang et al., 2021b). Reducing the dimension can decrease the invalid bands and autocorrelation caused by massive data input, to make the machine learning model more accurate and efficient. In addition, compared with other machine learning algorithms, RF has fewer parameters (Wang et al., 2016). Hence, by incorporating the optimal features of SIs and CWT, the RF model was significantly improved. These results suggest that incorporating suitable input variables could significantly improve model accuracy and robustness. In addition, to determine the stability of the model, independent validation for the RF model was also conducted. The results were similar to the cross-validation results.

In sum, the combination of spectral index, wavelet analysis, and machine learning algorithms provides an efficient method for improving the estimation accuracy of rice LPC. Our findings may be useful for real time monitoring and diagnosis of rice phosphorus nutrition, and to provide a basic guideline for the best management practices of rice P fertilizer in the future.

## 5 Conclusions

In this study, we integrated SIs and CWT of the original spectrum with machine learning algorithms to offer an optimal prediction model for rice P concentration. The SIs + CWT coupling with the RF model can significantly increase rice LPC estimation accuracy while significantly reducing the number of input variables. The prediction accuracy of LPC with R^2^ was increased by 32% compared with the linear regression models. This study provides a new perspective to effectively estimate the P concentration in rice leaves. However, this study only aimed at the tillering stage of potted rice. Hence, in order to improve the applicability and prediction accuracy of the model, more data fusion approaches and new machine learning methods should be considered.

## Data availability statement

The original contributions presented in the study are included in the article/**Supplementary Material.** Further inquiries can be directed to the corresponding author.

## Author contributions

NC and YZ designed the research and supervised the project. TW, TLW, and ZL performed research and analysed data. TW, YZ, and NC wrote and revised the manuscript. All authors contributed to the article and approved the submitted version.
