# Supplementary material for: Based on machine learning algorithms for estimating leaf phosphorus concentration of rice using optimized spectral indices and continuous wavelet transform
Source: Front Plant Sci. 2023 May 25;14:1185915. doi: 10.3389/fpls.2023.1185915 (PMC10251409; doi:10.3389/fpls.2023.1185915)
Supplement: Supplementary file 1 [file DataSheet_1.pdf]

frontiers

Supplementary Material

Based on machine learning algorithms for estimating leaf phosphorus concentration of rice using optimized spectral indices and continuous wavelet transform

Yi Zhang[#], Teng Wang[#], Zheng Li, Tianli Wang, Ning Cao[*]

*** Correspondence:** Corresponding Author: cao_ning@jlu.edu.cn

1 Supplementary Figures and Tables

1.1 Details of wavelet functions

Fifteen classic wavelet functions were used in this study, and more details were shown in Supplementary Table 1. The correlation between fifteen wavelet functions and the LPC of rice were shown in Supplementary Figure 1, and ten scales were calculated for each wavelet function. The R^2 ranged from 0.36-0.53, and the transformation effect based on the Mexh function produced the highest model accuracy ($R^2 > 0.5$). Therefore, the Mexh function was used as the optimal wavelet function to extract wavelet features.

Supplementary Table 1. Details of fifteen wavelet functions.

Wavelet function	Family	Orthogonal	Biorthogonal	Compact Support	Continuous Wavelet Transform	MATLAB® Command
bior3.5	Biorthogonal	No	Yes	Yes	Possible	waveinfo('bior')
cgau5	Cgau	No	No	No	Possible	waveinfo('cgau')
coif3	Coiflets	Yes	Yes	Yes	Possible	waveinfo('coif')
db10	Daubechies	Yes	Yes	Yes	Possible	waveinfo('db')
dmey	Dmeyer	Yes	Yes	Yes	Possible	waveinfo('dmey')
cmor1-0.5	Cmor	No	No	No	Possible	waveinfo('cmor')
fbsp2-1-0.5	Fbsp	No	No	No	Possible	waveinfo('fbsp')
gaus5	Gaus	No	No	No	Possible	waveinfo('gaus')
haar	Haar	Yes	Yes	Yes	Possible	waveinfo('haar')
mexh	Mexican Hat	No	No	No	Possible	waveinfo('mexh')
meyr	Meyer	Yes	Yes	No	Possible	waveinfo('meyr')
morl	Morlet	No	No	No	Possible	waveinfo('morl')
rbio4-4	ReverseBior	No	Yes	Yes	Possible	waveinfo('bior')
shan1-1	Shan	No	No	No	Possible	waveinfo('shan')
sym8	Symlets	Yes	Yes	Yes	Possible	waveinfo('sym')

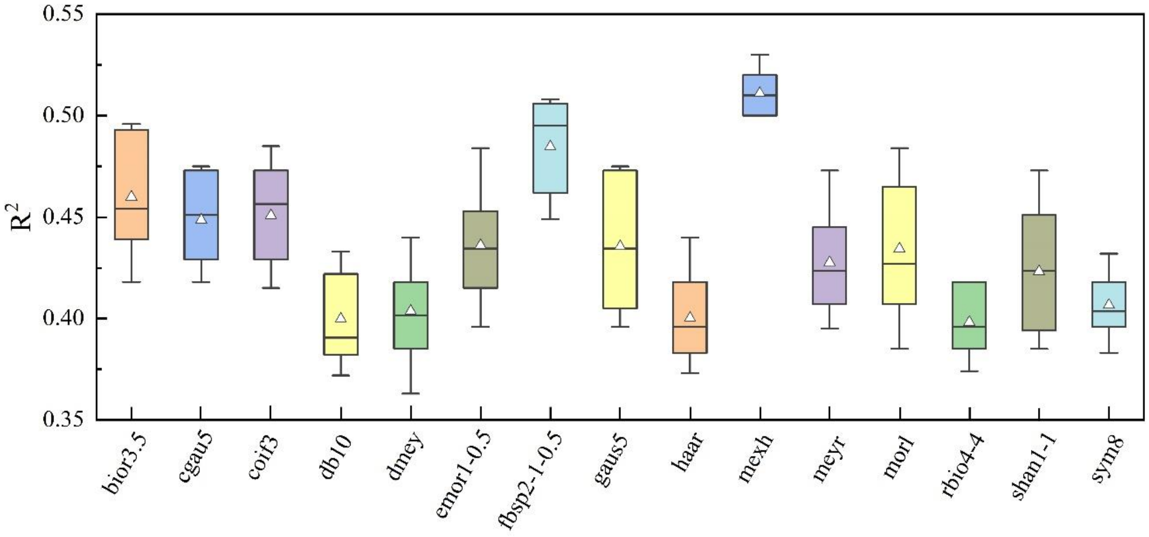

Supplementary Figure 1. Determination coefficient (R^2) between the wavelet functions and rice LPC.

1.2 Details of machine learning algorithms

In this study, the PLSR, LASSO, RF, SVM, and BPANN programs were applied using Python (version 3.7.0, The Python Software Foundation, USA) software, and the parameters were the default settings. Detail information were shown in Supplementary Table 2.

Supplementary Table 2. Details of five machine learning algorithms.

Machine learning algorithm	Parameter settings
PLSR	n_components=2 scale=True max_iter=500 tol=1×10^{-6} copy=True
LASSO	alpha=1.0 fit_intercept=True normalize=False precompute=False copy_X=True max_iter=1000 tol=0.0001 warm_start=False positive=False random_state=None selection='cyclic'
RF	n_estimators=100 criterion='mse' max_depth=None min_samples_split=2 min_samples_leaf=1 min_weight_fraction_leaf=0.0 max_features='auto' max_leaf_nodes=None min_impurity_decrease=0.0 min_impurity_split=None bootstrap=True oob_score=False n_jobs=None random_state=None verbose=0 warm_start=False ccp_alpha=0.0 max_samples=None
SVM	kernel='rbf' degree=3 gamma='scale' coef0=0.0 tol=0.001 C=1.0 epsilon=0.1 shrinking=True cache_size=200 verbose=False max_iter=-1

BPANN	hidden_layer_sizes= (100,) activation='relu' solver='adam' alpha=0.0001 batch_size='auto' learning_rate='constant' learning_rate_init=0.001 power_t=0.5 max_iter=200 shuffle=True random_state=None tol=0.0001 verbose=False warm_start=False momentum=0.9 nesterovs_momentum=True early_stopping=False validation_fraction=0.1 beta_1=0.9 beta_2=0.999 epsilon=1 $\times 10^{-8}$ n_iter_no_change=10 max_fun=15000

1.3 Details of input variables of machine learning models

The LPC of rice was taken as the dependent variable. The independent variables were the original full band (all 2151 bands ranging from 350-2500 nm, OR), optimized SIs (10 best features), optimized CWT (10 best features), and the combination of SIs and CWT (20 input features, SIs + CWT), respectively. Supplementary Table 3 shows the details of input variables.

Supplementary Table 3. Details of input variables for machine learning models.

Input variable	Variable feature	No. of features
OR	2151 bands ranging from 350-2500 nm	2151
SIs	DSI (1089, 1070 nm), DSI (1090, 1071 nm)<br>DSI (1090, 1072 nm), DSI (1091, 1072 nm)<br>DSI (1089, 1069 nm), RSI (1009, 990 nm)<br>RSI (1008, 991 nm), RSI (1004, 993 nm)<br>RSI (1002, 994 nm), RSI (999, 995 nm)	10
CWT	Mexh (1550 nm, 1), Mexh (982 nm, 2)<br>Mexh (983 nm, 3), Mexh (982 nm, 4)<br>Mexh (982 nm, 5), Mexh (1680 nm, 6)<br>Mexh (1679 nm, 7), Mexh (1679 nm, 8)<br>Mexh (982 nm, 9), Mexh (982 nm, 10)	10
SIs + CWT	DSI (1089, 1070 nm), DSI (1090, 1071 nm)<br>DSI (1090, 1072 nm), DSI (1091, 1072 nm)<br>DSI (1089, 1069 nm), RSI (1009, 990 nm)<br>RSI (1008, 991 nm), RSI (1004, 993 nm)<br>RSI (1002, 994 nm), RSI (999, 995 nm)<br>Mexh (1550 nm, 1), Mexh (982 nm, 2)<br>Mexh (983 nm, 3), Mexh (982 nm, 4)<br>Mexh (982 nm, 5), Mexh (1680 nm, 6)<br>Mexh (1679 nm, 7), Mexh (1679 nm, 8)<br>Mexh (982 nm, 9), Mexh (982 nm, 10)	20

1.4 Independent validation results of machine learning model (RF)

RF model had the best results of calibration and validation R^2 in cross-validation. To determine the stability of this machine learning model, independent validation for RF model was also conducted. Wuyoudao 4 was used for model training (n = 240), and another variety Longjing 31 was used for testing (n = 108). Supplementary Table 4 shows the independent validation results. RF algorithm fed with the combination of SIs and CWT (RF – SIs + CWT) improved estimation accuracy while significantly reducing the number of input variables. The results were similar to the results of cross-validation. In the validation set, R^2 and RMSE were 0.61 and 0.62 mg g^{-1}, respectively. And the model presents the lowest AIC of 4314.77.

Supplementary Table 4. Independent validation results of machine learning model (RF).

Variables	No. of bands or features	Calibration dataset	Validation dataset		AIC
		R^2	R^2	RMSE	
OR	2151	0.93	0.60	0.62	4315.34
SIs	10	0.92	0.53	0.63	4323.74
CWT	10	0.93	0.55	0.63	4322.42
SIs + CWT	20	0.93	0.61	0.62	4314.77
